# Supplementary material for: Global conservation status of the jawed vertebrate Tree of Life
Source: Nat Commun. 2024 Feb 29;15:1101. doi: 10.1038/s41467-024-45119-z (PMC10904806; doi:10.1038/s41467-024-45119-z)
Supplement: Supplementary file 1 — Supplementary Information [file 41467_2024_45119_MOESM1_ESM.pdf]

Supplementary information

Global conservation status of the jawed vertebrate Tree of Life

Gumbs et al.

Contents

Supplementary Results: Using taxonomy to identify candidate EDGE species.

Supplementary Figure 1: Total and threatened evolutionary history of jawed vertebrate clades.

Supplementary Figure 2: Unique evolutionary history of jawed vertebrate species.

Supplementary Figure 3: Evolutionarily Distinct and Globally Endangered (EDGE) scores for jawed vertebrate clades.

Supplementary Figure 4: Relationship between Evolutionarily Distinct and Globally Endangered (EDGE) Lineages and EDGE Species rankings.

Supplementary Figure 5: Evolutionary ages of families across jawed vertebrate clades.

Supplementary Table 1: Status of families across jawed vertebrate clades.

Supplementary References.

Supplementary Results

Using taxonomy to identify candidate EDGE species

To enable the identification of candidate EDGE species from clades lacking sufficient phylogenetic data for a complete EDGE assessment, we explored whether taxonomy and extinction risk information alone could reliably inform the designation of priority EDGE species. All monotypic threatened families of birds, lepidosaurs, mammals, chondrichthyans, testudines and crocodylians are priority EDGE species (27 families). Of the five monotypic threatened families of ray-finned fish, two are priority EDGE species. The other three ray-finned fish families that are not EDGE priorities are either too uncertain in their phylogenetic position or have insufficient EDGE scores to qualify as EDGE species. All monotypic families that are EN or higher on the IUCN Red List are priority EDGE species.

For families with two or more species that are all threatened (21 families), 85.2% (121 of 142 spp.) of constituent species are EDGE species. This increases to 94.4% (100 of 106 spp.) when just EN, CR and Extinct in the Wild (EW) species are included, and to 100% (44 of 44 spp.) when just CR and EW are considered. Similarly, for species in families where all assessed species are threatened but may be lacking data sufficient assessments for at least one family member (42 families), 90.8% (139 of 153 spp.) of EN, CR and EW species are EDGE priorities. This figure increases to 95.7% (66 of 69 spp.) when considering only CR and EW species. This is compared with 43.5% of all threatened species, 57.1% of EN, CR and EW species, and 72% of CR and EW species only when family species richness and proportion of threat in the family are not taken into consideration (i.e., across all threatened jawed vertebrate species).

Our results suggest that, for clades lacking sufficient information to generate EDGE priorities, we can rely instead on taxonomic information; for example, monotypic families that are Endangered, Critically Endangered, or Extinct in the Wild are highly likely to comprise candidate EDGE species. In addition, for polytypic families where all species (or all assessed species) are threatened, species listed as Critically Endangered or Extinct in the Wild likely also be candidate EDGE species. As the application of machine learning approaches to predict extinction risk grows[1,2], these data may also inform the identification of highly evolutionarily distinct species in need of conservation action.

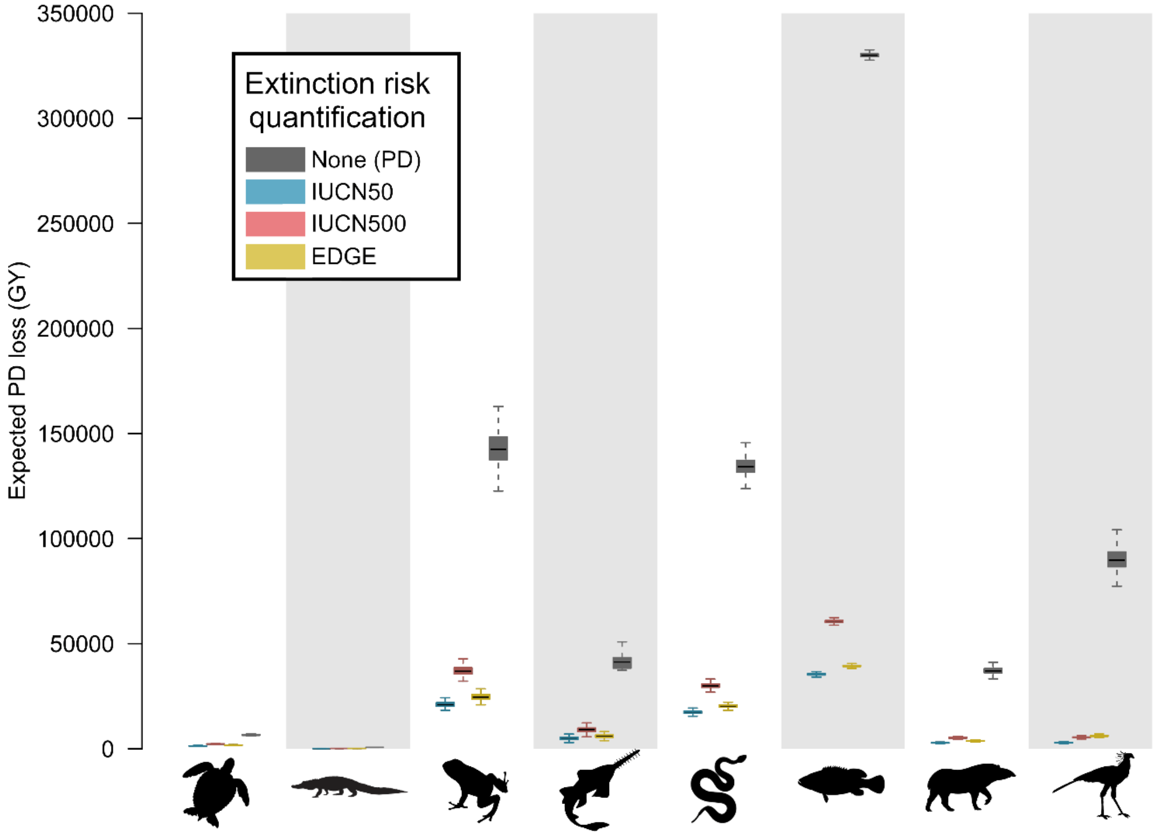

Supplementary Figure 1: Total and threatened evolutionary history of jawed vertebrate clades. The total evolutionary history (grey, median total phylogenetic diversity (PD) of each group) and threatened evolutionary history, in billions of years (giga years, GY) for three extinction risk quantifications: blue = 'IUCN50', a 50-year extrapolation of extinction risk from IUCN Red List Criterion E; red = 'IUCN500', a 500-year extrapolation of extinction risk from IUCN Red List Criterion E; yellow = 'EDGE', the extinction risk weighting used to generate priority EDGE Lists under the EDGE2 protocol and underpinning the proposed Phylogenetic Diversity indicator for the Kunming-Montreal Global Biodiversity Framework (see Methods for extinction risk values used). Boxplot centre line shows the median; box limits, upper and lower quartiles; whiskers show 1.5x interquartile range across 1,000 values. Clades from left to right: testudines (351 spp.); crocodylians (25 spp.); amphibians (8,024 spp.); chondrichthyans (1,290 spp.); lepidosaurs (10,735 spp.); ray-finned fish (32,760 spp.); mammals (6,253 spp.); and birds (10,988 spp.). Source data are provided as a Source Data file.

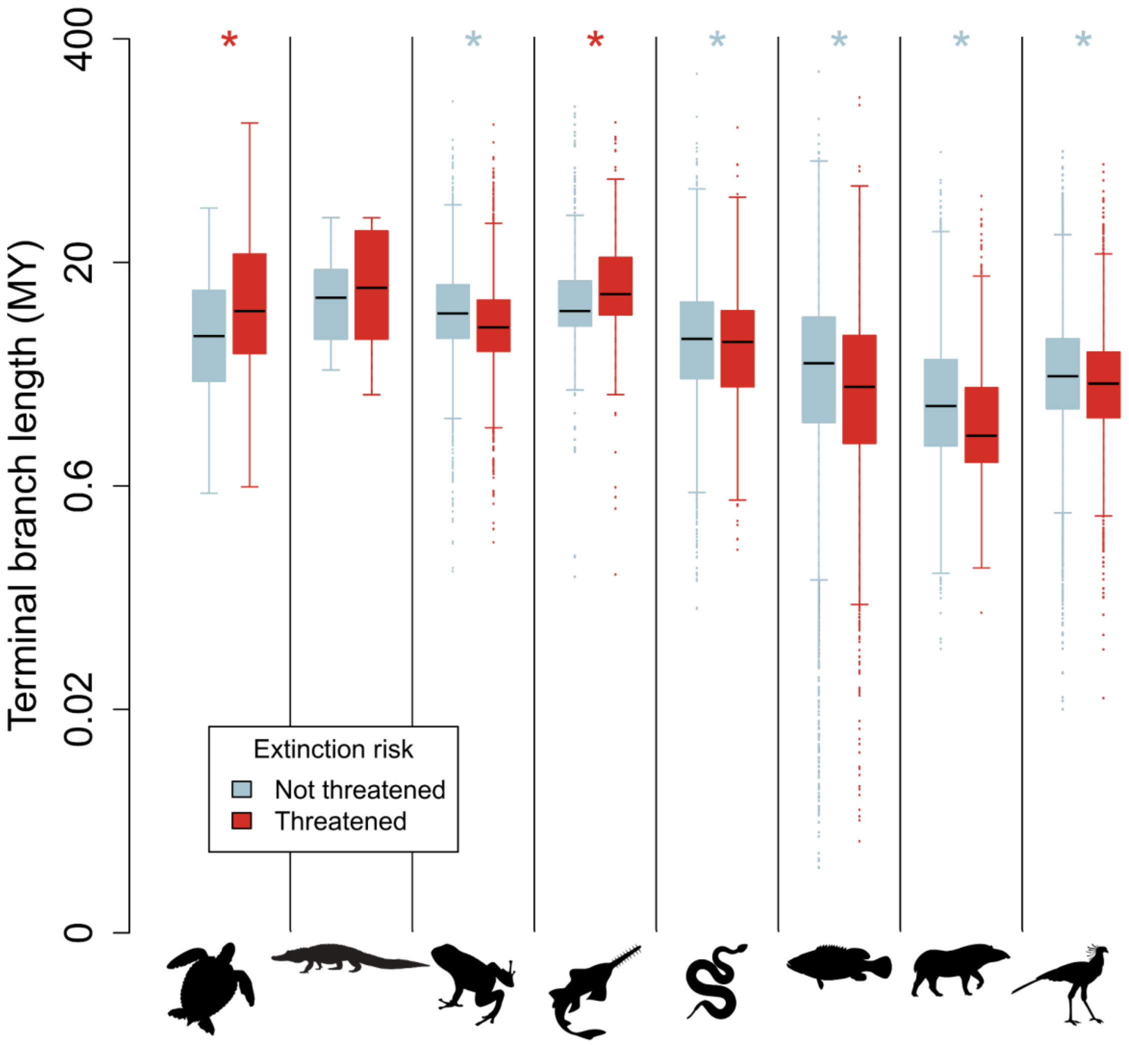

Supplementary Figure 2: Unique evolutionary history of jawed vertebrate species. The median terminal branch length (TBL; log-scale) of threatened (Vulnerable, Endangered, Critically Endangered on the IUCN Red List; red) and non-threatened (blue) species across jawed vertebrate clades, measured in millions of years (MY). Blue asterisks indicate that non-threatened species have significantly more unique evolutionary history than threatened species. Red asterisks indicate that threatened species have significantly more unique evolutionary history than non-threatened species. No asterisk indicates no difference between the unique evolutionary history embodied by threatened and non-threatened species. Statistical significance from a two-sample t-test with alpha of 0.05. Boxplot centre line shows the median; box limits, upper and lower quartiles; whiskers show 1.5x interquartile range, across 1,000 values for each clade. Source data are provided as a Source Data file.

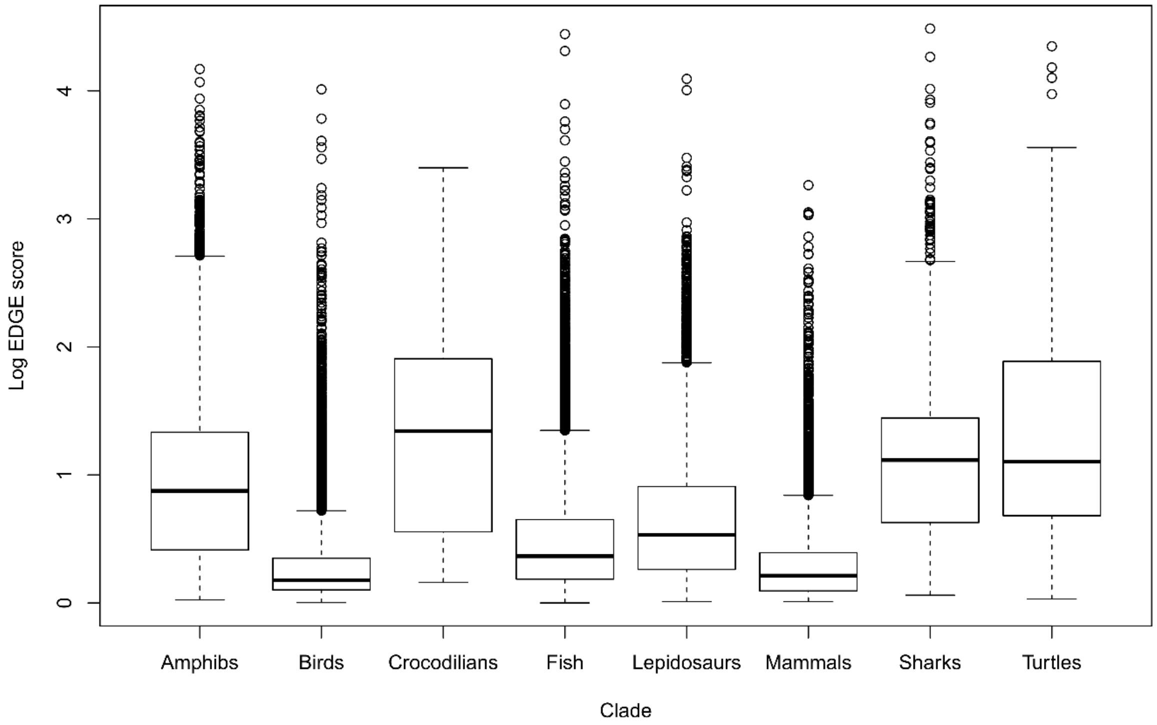

Supplementary Figure 3: Evolutionarily Distinct and Globally Endangered (EDGE) scores for jawed vertebrate clades. The distribution of median EDGE scores for each species across jawed vertebrate clades. Boxplot centre line shows the median; box limits, upper and lower quartiles; whiskers show 1.5x interquartile range. Source data are provided as a Source Data file.

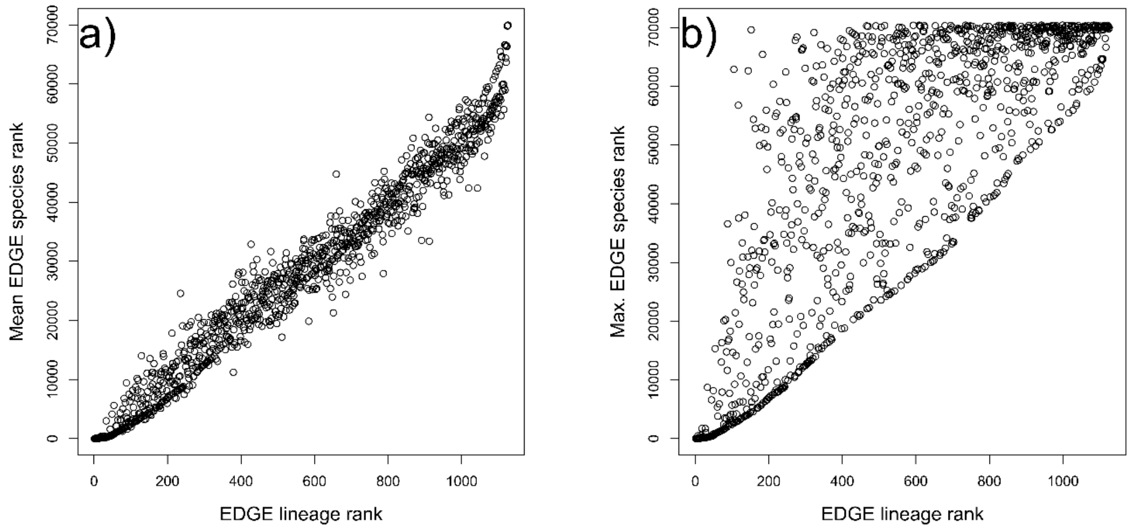

Supplementary Figure 4: Relationship between Evolutionarily Distinct and Globally Endangered (EDGE) Lineages and EDGE Species rankings. The (a) relationship between family-level EDGE ranking and the average EDGE ranking of the constituent species for each family, across all jawed vertebrate clades; and (b) relationship between family richness and the average EDGE ranking of the constituent species of each family, across all jawed vertebrate clades. Source data are provided as a Source Data file.

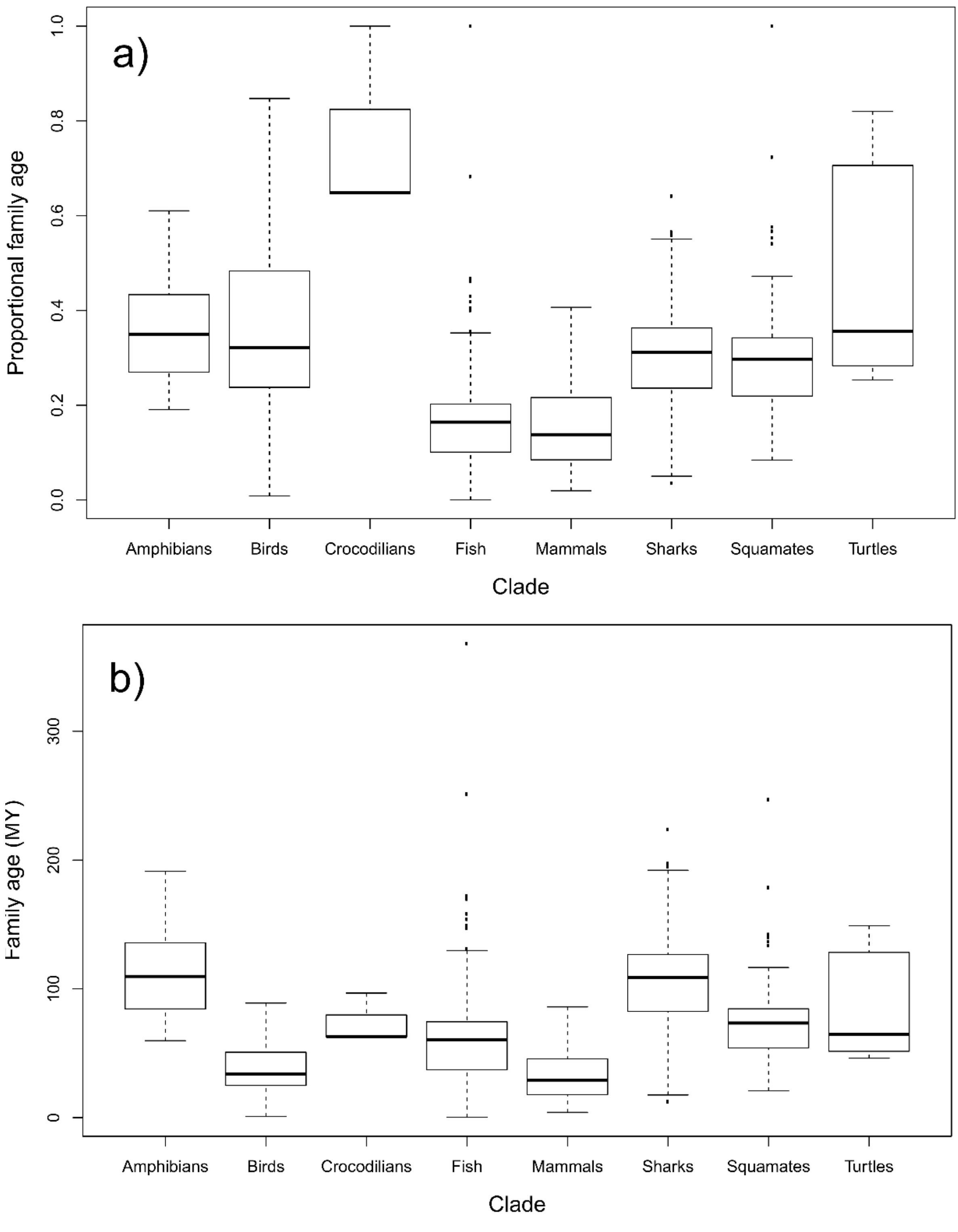

Supplementary Figure 5: Evolutionary ages of families across jawed vertebrate clades. The distribution of a) the age of families, proportionate to the overall age of the clade; and b) the raw age of families, across jawed vertebrate clades. MY = millions of years. Boxplot centre line shows the median; box limits, upper and lower quartiles; whiskers show 1.5x interquartile range. Source data are provided as a Source Data file.

Supplementary Table 1: Status of families across jawed vertebrate clades. The number and proportion of threatened families across jawed vertebrate clades.

Clade	*Number of families*					
	Total		*With at least one threatened species and no species known to be non-threatened*			
	All	*Monotypic*	*All*		monotypic	
			Fully data sufficient	*Some data insufficient*	*Fully data sufficient*	
			(% of all families)	*(% of all families)*	*(% of all monotypic families)*	*(% of all data sufficient monotypic families)*
Amphibians	76	1	3 (3.9%)	4 (5.3%)	0 (0%)	0 (0%)
Birds	234	34	9 (3.7%)	9 (3.7%)	5 (14.7%)	5 (14.7%)
Crocodilians	3	1	1 (33.3%)	1 (33%)	1 (100%)	1 (100%)
Lepidosaurs	75	8	3 (4%)	4 (5.3%)	3 (37.5%)	3 (42.9%)
Mammals	160	28	20 (12.5%)	25 (15.6%)	11 (39.3%)	11 (39.3%)
Ray-finned fish	491	58	7 (1.4%)	14 (2.9%)	5 (8.6%)	5 (16.7%)
Sharks and rays	64	11	6 (9.4%)	11 (17.2%)	3 (27.3%)	3 (30%)
Turtles	14	4	4 (28.6%)	6 (42.9%)	4 (100%)	4 (100%)
All	1,126	145	53 (4.7%)	74 (6.6%)	32 (22.1%)	32 (27.8%)

Supplementary References

1. Caetano, G. H. de O. *et al.* Automated assessment reveals that the extinction risk of reptiles is widely underestimated across space and phylogeny. *PLoS Biol* **20**, e3001544 (2022).
2. Borgelt, J., Dorber, M., Høiberg, M. A. & Verones, F. More than half of data deficient species predicted to be threatened by extinction. *Commun Biol* **5**, 679 (2022).
