# Supplementary material for: Global conservation status of the jawed vertebrate Tree of Life
Source: Nat Commun. 2024 Feb 29;15:1101. doi: 10.1038/s41467-024-45119-z (PMC10904806; doi:10.1038/s41467-024-45119-z)
Supplement: Supplementary file 3 — Description of Additional Supplementary Files [file 41467_2024_45119_MOESM3_ESM.pdf]

Description of Additional Supplementary Files

File Name: Supplementary Data 1
Description: Species-level, familylevel, and clade-level data for all jawed vertebrates included in this study.
