# Supplementary material for: Global conservation status of the jawed vertebrate Tree of Life
Source: Nat Commun. 2024 Feb 29;15:1101. doi: 10.1038/s41467-024-45119-z (PMC10904806; doi:10.1038/s41467-024-45119-z)
Supplement: Supplementary file 5 — Reporting Summary [file 41467_2024_45119_MOESM5_ESM.pdf]

nature portfolio

Corresponding author(s): Rikki Gumbs

Last updated by author(s): 13/12/23

Reporting Summary

Nature Portfolio wishes to improve the reproducibility of the work that we publish. This form provides structure for consistency and transparency in reporting. For further information on Nature Portfolio policies, see our Editorial Policies and the Editorial Policy Checklist.

Statistics

For all statistical analyses, confirm that the following items are present in the figure legend, table legend, main text, or Methods section.

n/a	Confirmed	
☐	☒	The exact sample size (*n*) for each experimental group/condition, given as a discrete number and unit of measurement
☐	☒	A statement on whether measurements were taken from distinct samples or whether the same sample was measured repeatedly
☐	☒	The statistical test(s) used AND whether they are one- or two-sided<br>*Only common tests should be described solely by name; describe more complex techniques in the Methods section.*
☒	☐	A description of all covariates tested
☐	☒	A description of any assumptions or corrections, such as tests of normality and adjustment for multiple comparisons
☐	☒	A full description of the statistical parameters including central tendency (e.g. means) or other basic estimates (e.g. regression coefficient) AND variation (e.g. standard deviation) or associated estimates of uncertainty (e.g. confidence intervals)
☐	☒	For null hypothesis testing, the test statistic (e.g. F, t, r) with confidence intervals, effect sizes, degrees of freedom and P value noted<br>*Give P values as exact values whenever suitable.*
☒	☐	For Bayesian analysis, information on the choice of priors and Markov chain Monte Carlo settings
☒	☐	For hierarchical and complex designs, identification of the appropriate level for tests and full reporting of outcomes
☒	☐	Estimates of effect sizes (e.g. Cohen's *d*, Pearson's *r*), indicating how they were calculated

Our web collection on statistics for biologists contains articles on many of the points above.

Software and code

Policy information about availability of computer code

Data collection	No software was used
Data analysis	Species-specific (terminal branch length, EDGE) and clade-level (expected loss) scores were generated using the 'EDGE2' code available here: https://github.com/rgumbs/EDGE2

For manuscripts utilizing custom algorithms or software that are central to the research but not yet described in published literature, software must be made available to editors and reviewers. We strongly encourage code deposition in a community repository (e.g. GitHub). See the Nature Portfolio guidelines for submitting code & software for further information.

Data

Policy information about availability of data

All manuscripts must include a data availability statement. This statement should provide the following information, where applicable:

- Accession codes, unique identifiers, or web links for publicly available datasets
- A description of any restrictions on data availability
- For clinical datasets or third party data, please ensure that the statement adheres to our policy

The data underlying all results, figures and tables are available on Figshare (DOI: 10.6084/m9.figshare.22689793) and source data are provided with this paper. Summary statistics of species, family and clade-level evolutionary history and EDGE scores are provided as part of Supplementary Data 1. Species taxonomies were taken from Frost's Amphibian Species of the World (https://amphibiansoftheworld.amnh.org/), The Reptile Database (http://www.reptile-database.org/data/),

BirdLife International (https://datazone.birdlife.org/species/taxonomy), the ASM Mammal Diversity Database (https://www.mammaldiversity.org/), and Fishbase (https://www.fishbase.se/search.php). Phylogenies were taken from VertLife (https://vertlife.org/data/) and the Fish Tree of Life (https://fishtreeoflife.org/). Extinction risk data were taken from the IUCN Red List version 2021.1 (https://www.iucnredlist.org/).

Research involving human participants, their data, or biological material

Policy information about studies with human participants or human data. See also policy information about sex, gender (identity/presentation), and sexual orientation and race, ethnicity and racism.

Reporting on sex and gender	NA
Reporting on race, ethnicity, or other socially relevant groupings	NA
Population characteristics	NA
Recruitment	NA
Ethics oversight	NA

Note that full information on the approval of the study protocol must also be provided in the manuscript.

Field-specific reporting

Please select the one below that is the best fit for your research. If you are not sure, read the appropriate sections before making your selection.

☐ Life sciences ☐ Behavioural & social sciences ☒ Ecological, evolutionary & environmental sciences

For a reference copy of the document with all sections, see nature.com/documents/nr-reporting-summary-flat.pdf

Ecological, evolutionary & environmental sciences study design

All studies must disclose on these points even when the disclosure is negative.

Study description	Calculated the expected loss of evolutionary history and family-level and species-level scores of expected loss that could be averted (EDGE scores) for amphibians, birds, mammals, reptiles, sharks and rays, and ray-finned fish.
Research sample	All described species of amphibians, birds, mammals, reptiles, sharks and rays, and ray-finned fish.
Sampling strategy	We utilised a distribution of 1,000 phylogenies sampled at random from a larger 10,000 phylogeny distribution for each clade to calculate data.
Data collection	Phylogenetic, extinction risk and taxonomic data were collected by the authors for all species from open sources online
Timing and spatial scale	All data were collected between July 2020 the publication of the 2021.1 version of the IUCN Red List, and are global in scale.
Data exclusions	No data were excluded
Reproducibility	All data sources and procedures are described in sufficient detail to enable all results to be reproducible, with data underlying all results presented provided for validation.
Randomization	Null distributions used for comparison with observed data were generated by selecting sets of species at random, with the sets selected to match (and control for) the observed taxonomic and extinction risk distribution of species sets of interest.
Blinding	NA

Did the study involve field work? ☐ Yes ☒ No

Reporting for specific materials, systems and methods

We require information from authors about some types of materials, experimental systems and methods used in many studies. Here, indicate whether each material, system or method listed is relevant to your study. If you are not sure if a list item applies to your research, read the appropriate section before selecting a response.

Materials & experimental systems

n/a	Involved in the study
☒	☐ Antibodies
☒	☐ Eukaryotic cell lines
☒	☐ Palaeontology and archaeology
☒	☐ Animals and other organisms
☒	☐ Clinical data
☒	☐ Dual use research of concern
☒	☐ Plants

Methods

n/a	Involved in the study
☒	☐ ChIP-seq
☒	☐ Flow cytometry
☒	☐ MRI-based neuroimaging
